# Parental predictors of childhood vaccination adherence in border areas of Southern Vietnam: a first look at minority communities

**DOI:** 10.1016/j.jped.2025.04.005

**Published:** 2025-05-21

**Authors:** An Dai Tran, Charuai Suwanbamrung, Muhammad Haroon Stanikzai, Nirachon Chutipattana, Shamarina Shohaimin, Patthanasak Khammaneechan, Le Minh Luan, Tran Phu Dien, Truong Thanh Nam, Phan Thanh Tung, Cua Ngoc Le

**Affiliations:** a Walailak University, School of Public Health, Public Health Research Program, Nakhon Si Thammarat, Thailand; b Dong Thap Provincial Center for Disease Control, Dong Thap, Vietnam; c Walailak University, Excellent Center for Public Health Research (EC for PHR), Nakhon Si Thammarat, Thailand; d Kandahar University, Faculty of Medicine, Department of Public Health, Kandahar, Afghanistan; e Universiti Putra Malaysia, Faculty of Science, Department of Biology, Selangor, Malaysia; f Dong Thap Medical College, Dong Thap, Vietnam; g Can Tho University of Medicine and Pharmacy, Faculty of Public Health, Can Tho City, Vietnam

**Keywords:** Immunization coverage, Parents/psychology, Health belief model, Minority groups

## Abstract

**Objectives:**

Suboptimal timeliness and coverage of childhood vaccination programs undermined their effectiveness in achieving population-level immunity. This issue is particularly concerning among minority populations, where disparities in vaccination adherence persist. To address this gap, the study assessed the extent of parental adherence to age-appropriate childhood vaccination and its predictors among the minority children under five years of age.

**Methods:**

This cross-sectional study was conducted in three districts of Dong Thap Province, Vietnam, and neighboring Cambodia. A total of 449 ethnic minority parents with children under five years old participated. Data were gathered through face-to-face household interviews using a structured questionnaire, complemented by direct observation of the children’s vaccination cards to verify adherence. Binary logistic regression was used to identify predictors of vaccination adherence.

**Results:**

The adherence rate to childhood vaccination among children in the minority population was 18.9 %. Parental adherence was significantly higher for children under one year of age (aOR = 2.54, 95 % CI: 1.29–5.03) and for firstborn children (aOR = 3.48, 95 % CI: 1.36–9.92). Within the Health Belief Model framework, greater perceived barriers were associated with lower adherence (aOR = 0.32, 95 % CI: 0.21–0.49), while higher parental self-efficacy was linked to increased adherence (aOR = 1.84, 95 % CI: 1.11–3.11).

**Conclusion:**

This study revealed a low parental adherence rate (18.9 %) to childhood vaccination. A child’s age, birth order, perceived barriers, and parental self-efficacy influenced adherence. These findings emphasize the need to incorporate these factors into targeted policies and interventions for improving immunization rates in minority populations and comparable settings.

## Introduction

The Expanded Program on Immunization (EPI) has played a key role in global health, contributing to the eradication of smallpox and the elimination of polio and neonatal tetanus. As a national initiative, EPI provides free vaccines, while the immunization schedule specifies the timing and sequence needed for full protection. These achievements align with Sustainable Development Goals (SDG) 3 (Health) and SDG 10 (Reduced Inequalities).^1^ Globally, EPI prevents 2–3 million deaths annually;[1] in Vietnam, it averted an estimated 5.7 million disease cases and 26,000 deaths between 1980 and 2010.^2^

However, ethnic minorities and other vulnerable groups still face barriers to vaccination, including poverty, limited education, and remote geography.^3^ In Vietnam, third-dose DPT-Hep B-Hib and first-dose MCV coverage in 2023 were 65 % and 82 %, respectively.^4^ Children in rural and ethnic minority communities often receive delayed or incomplete vaccinations.^5^ According to the study conducted in 2008 in The Netherlands, 35 % of Orthodox Protestant children were unvaccinated;[3] in 2017, along the Thailand–Myanmar border, coverage for DTP, HBV, OPV, and measles vaccines ranged from 54.6 % to 56.3 %.^6^ These gaps increase the risk of outbreaks, threatening both minority groups and the general population.^7^

This study adopts the Health Belief Model (HBM) to examine factors influencing vaccination adherence among minority communities. HBM suggests that health behaviors are shaped by perceived susceptibility, severity, benefits, barriers, and cues to action.^8^ Since parental beliefs are central to vaccination decisions, applying HBM provides valuable insights into how these perceptions affect adherence to the EPI.^9^

Despite EPI’s global success in improving child health, gaps remain in addressing the needs of minority populations facing complex barriers—such as poverty, language, and geographic isolation—which are often underrepresented in research.^3^,^6^,^10^ In Vietnam, national reports rarely disaggregate vaccine coverage data by minority status, and studies often overlook timeliness, focusing only on overall coverage.^11^ A study found significantly lower rates of timely vaccination among rural and minority children, emphasizing the need for Vietnam’s EPI to prioritize timeliness in reaching these underserved groups.^5^ Yet, the critical factor in building immunity remains underexplored in current research.^10^,^12^

Beyond timeliness issues, disparities in access to immunization services worsen vaccination inequities. Disadvantaged groups in Vietnam, particularly ethnic minorities, face significant barriers - including language limitations that hinder understanding of immunization messages.^13^ In Dong Thap Province, Khmer and Cham communities encounter additional challenges such as cross-border migration, sociocultural beliefs about disease, and limited healthcare infrastructure, all of which impede timely and equitable vaccination.

Although parental decisions impact vaccination adherence, they are shaped by broader systemic factors like healthcare access and vaccine availability. Addressing these structural barriers is key to improving vaccination rates and equity among minority populations. This study hypothesized that socio-demographic factors, HBM constructs, vaccine provision, and healthcare accessibility predict adherence among ethnic minority parents in Dong Thap Province, Vietnam. Therefore, this study aimed to determine levels of parental adherence to EPI and identify key predictors of vaccination adherence among ethnic minorities in Dong Thap province, Vietnam. The findings aim to inform practical strategies for improving vaccination timeliness and equity in this vulnerable group.

## Methodology

### Study design and setting

This cross-sectional study was conducted from August 2023 to June 2024, targeting ethnic minority communities in three border districts of Dong Thap Province, Vietnam. These areas face vaccination challenges due to high mobility from cross-border migration for work and trade, complicating timely immunization tracking.

### Participant selection and sampling

$$n=Z^{2}1-\alpha /2\frac{p\left(1-p\right)}{d^{2}}$$

The sample size was calculated using the single population proportion formula, assuming 50 % adherence (unknown adherence proportion), a 95 % confidence level (*Z* = 1.96), and ±5 % absolute precision.^14^ This yielded 384 participants, increased by 10 % to account for nonresponse, totaling 422. Inclusion criteria included ethnic minority parents or caregivers of children under five, residing in Dong Thap, and consenting to participate. Exclusion criteria were refusal, relocation, or incomplete responses.

A multi-stage sampling method was used. First, three districts (Tan Hong, Hong Ngu, and Hong Ngu City) were purposively selected due to their proximity to Cambodia and high ethnic minority populations (mainly Khmer and Cham). All 26 communes in these districts were treated as clusters. From each commune, 17 eligible households were randomly selected using the citizen management list—a local government database containing household demographic and residency data, accessed through local authorities to ensure accuracy.

To confirm whether selected households met the inclusion criteria, the research team verified the ethnicity of household members through self-report and cross-checked with the citizen management list before the survey administration

Of 470 eligible parents approached, 460 agreed to participate; 11 were later excluded due to incomplete responses, resulting in a final sample of 449. The study targeted ethnic minority parents across three border districts in Dong Thap Province, capturing diverse geographic and socio-demographic characteristics. Despite using a multi-stage random sampling method, selection bias may exist due to non-response and excluded data, potentially affecting representativeness if these groups differed in adherence behaviors or healthcare access.

### Research instrument and data collection

#### Questionnaire

A literature review of studies on parental vaccine intention, acceptance, and adherence revealed various question types, including required vaccines, logistics, accessibility, perceptions, beliefs, and attitudes toward vaccination policies. Questions unrelated to this study (e.g., policy satisfaction or opinions on new policies) were excluded [15, 16, 17]. The final questionnaire comprised six parts, detailed in Appendix 1.

Adherence: Defined as receiving all vaccines within the recommended timeframe in Table A7/Appendix 3.^18^ Clarifications are in Appendix 1, Part 6.

Non-adherence: Defined as (i) missing any scheduled vaccine; (ii) receiving a dose beyond allowable delay (HBV: >24 hrs post-birth; others: >30 days late);^19^(iii) compounded delays affecting subsequent doses; or (iv) system-related delays (e.g., stockouts), which are recorded but not counted as parental non-adherence.

To ensure quality, the questionnaire’s content validity was reviewed by three experts. It achieved acceptable scale-level CVIs (S-CVI/Ave and S-CVI/UA > 0.80),^20^,^21^ with calculation details in Table A8/Appendix 3.

### Data collection

Data was collected between March and May 2024 by six village health volunteers (VHVs) trained to conduct face-to-face interviews using a structured questionnaire. The VHVs visited participants' homes, obtained permission, and recorded vaccination information from the child’s vaccination card. The survey took 20–30 min, during which participants could ask questions.

### Data analysis

#### Descriptive analysis

The proportion of adherence to the EPI was determined, providing a baseline understanding of the data.

### Content validity and reliability of the questionnaire

The content validity indices S-CVI/Ave and S-CVI/UA were calculated at 0.96 and 0.9, respectively, indicating satisfactory content validity (Details provided in Table A.8/Appendix 3). Additionally, the questionnaire demonstrated acceptable internal consistency, with a Cronbach’s alpha of 0.741 for items measuring parental perceptions of the EPI.

### Inferential analysis

A two-stage approach was employed:

Stage 1: Variable Screening

Socio-demographic characteristics of parents, vaccine provision, health service accessibility, and children’s demographic characteristics, were screened using Chi-square tests. When the expected cell counts were less than 5, Fisher’s exact test was applied. For parental perceptions in HBM, which were continuous variables, the Kolmogorov-Smirnov test confirmed non-normal distribution (*p* < 0.05). Therefore, the Mann-Whitney U test was applied to compare mean ranks of HBM construct scores between adherence and non-adherence groups. The purpose of this stage choose variables significantly associated with adherence (*p* < 0.05). The reduction of the number of independent variables entered into the logistic regression minimized the risk of overfitting the model.

Stage 2: Logistic Regression

Significant variables from stage 1 were included simultaneously in a binary logistic regression model to maintain theoretical integrity and avoid model instability caused by stepwise methods. The logistic regression model estimated adjusted odds ratios (aORs) with 95 % confidence intervals (CIs) to measure the association between predictors and adherence. aORs are derived from exponentiating β coefficients.

Assumptions of logistic regression: (i) Independence of observations was ensured as the dataset was drawn by a multi-stage random sampling approach. (ii) No multicollinearity: all included variables met the VIF < 3 and Tolerance > 0.2, (iii) Standardized residuals, Cook’s distance, and leverage values were examined, and no potential influential observations or extreme outliers were found.

The Hosmer-Lemeshow goodness-of-fit test was used to assess the model fit (*p* > 0.05). To evaluate how effectively the logistic regression model classified adherence, a Receiver Operating Characteristic (ROC) curve was plotted (1-specificity vs. sensitivity) to assess classification performance at different probability thresholds. Additionally, the Area Under the Curve (AUC) was used to measure how well the model distinguished between adherence and non-adherence.

The logistic regression equation is under the form. X1, X2,…Xk are significant variables in stage 1.

Log-odds of adherence (log p/1-p) = Constant + b0 + b1 × 1 + b2 × 2 + …+ bkXk

The predicted probability of parental vaccination adherence (p) was calculated by converting log odds to the probability.

All statistical analyses were performed using IBM SPSS software version 20.0.

### Ethics statement and consent to participate

The study protocol adhered to the principles outlined in the Declaration of Helsinki and received approval from the Human Research Ethics Committee of Walailak University (WU-EC-PU-0–017–67). Parents or legal guardians of children under the age of five provided written informed consent.

## Results

### The extent of parental adherence to EPI among the minority population

Among 449 participants, vaccination adherence was 18.9 % (95 % CI: 15.4 %–22.9 %), indicating low rates of age-appropriate immunization (see Figure A.1/Appendix 4). Timeliness varied across vaccines. JE1 (12 months) showed lower adherence, possibly due to prioritization of MCV at 9 months and limited awareness of JE’s importance. JE2 (18 months) had higher adherence, likely due to being administered alongside other boosters (MR, DPT). In contrast, JE3 (2 years) dropped sharply, as it falls outside core schedules, making it more easily missed. Reduced follow-up and perceived risk at age two may also contribute (Table 1).

**Table 1:** Timeliness of vaccination adherence by visit and vaccine type.

Visits	Vaccine	Adherence *n* (%)	Total *n*
At birth	BCG	385(85.7)	449
At birth	HBV	384(85.5)	449
2 months	DPT-Hep B-Hib 1	270 (61.2)	441
2 months	OPV 1	262 (59.5)	440
3 months	DPT-Hep B-Hib 2	293 (68.9)	425
3 months	OPV 2	294(69.3)	424
4 months	DPT-Hep B-Hib 3	257 (64.6)	398
4 months	OPV 3	246(62.8)	392
5 months	IPV	127(32.6)	389
9 months	MCV	161(44.6)	361
12 months	JE 1	159(49.1)	324
18 months	JE 2	226(71.5)	316
18 months	MR	109(38.5)	283
18 months	DPT	88(31.2)	282
2 years	JE 3	95(45.9)	207

### Bivariate analysis between vaccination adherence and the socio-demographic characteristics of parents and children, vaccine provision, accessibility of health service

#### Socio-demographic characteristics of parents

Most of the participants were females (86.2 %), mothers (64.1 %), aged 26–40 years (55.0 %), and lived in rural areas (68.2 %). Monthly income data showed that 53.0 % of respondents earned between 5000,000 and 10,000,000 Vietnamese Dong (VND). Occupationally, 44.3 % were housewives, followed by freelancers (19.6 %) (Details in Table A.1/Appendix 3).

#### Demographic characteristics of children

Children were mainly 2–5 years old (52.8 %) and had their birth weights from 2500 to 3000 *g* (50.1 %) (Details in Table A.2/Appendix 3).

Vaccine provision and accessibility to health service

In terms of vaccine provision, 57.2 % reported availability for their children, while 54.8 % reported adverse events. Regarding accessibility of health services, 94.0 % used a private motorbike to reach immunization facilities, with 64.6 % living from one to five kilometers away.

In summary, statistically significant variables such as participants' age (*p* < 0.001), living area (*p* = 0.003), income (*p* = 0.034), child's age, and birth order (*p* < 0.001) were included in the binary logistic regression model for determining predictors of vaccination adherence (Details in Table 2).

**Table 2:** Association between vaccination adherence and the socio-demographic characteristics of parents and children, vaccine provision, and accessibility of health service.

**Variables**		***n* ( %)**			
		**Total *n*=449**	**Adherence to EPI (*n*=85)**	**Non-adherence to EPI (*n*=364)**	***p***
Kinship to children	Father	41 (9.1)	4 (4.7)	37 (10.2)	0.105^a^
	Mother	288 (64.1)	64 (75.3)	224 (61.5)	
	Relative	7 (1.6)	1(1.2)	6 (1.6)	
	Grandparents	113 (25.2)	16(18.8)	97 (26.6)	
Parents’ gender	Male	62 (13.8)	7(8.2)	55 (15.1)	0.116
	Female	387 (86.2)	78(91.8)	309 (84.9)	
Living area	Urban	143 (31.8)	39 (45.9)	104 (28.6)	0.003
	Rural	306 (68.2)	46(54.1)	260(71.4)	
Family size	≤4 members	182 (40.5)	39 (45.9)	143 (39.3)	0.272
	>4 members	267 (59.5)	46 (54.1)	221 (60.7)	
Education level	Below senior high school	300 (66.8)	50 (58.8)	250 (68.7)	0.082
	Senior high school and upper	149 (33.2)	35 (41.2)	114 (31.3)	
Income	<5000,000 VND	123 (27.4)	14(16.5)	109 (29.9)	0.034
	5000,000 to 10,000,000 VND	238 (53.0)	54(63.5)	184 (50.5)	
	>10,000,000 VND	88 (19.6)	17(20)	71 (19.5)	
Occupation	Monthly salary jobs	54 (12.0)	10 (11.8)	44 (12.1)	0.595
	Freelance, seasonal work	88 (19.6)	12 (14.1)	76 (20.9)	
	Housewife	199 (44.3)	39 (45.9)	160 (44)	
	Student	68 (15.1)	13 (15.3)	55 (15.1)	
	Retirement	22 (5.1)	6 (7.1)	17 (4.7)	
	Farmer	17 (3.8)	5 (5.9)	12 (3.3)	
Parents’ age (year)	≤25	59 (13.1)	23 (27.1)	36 (9.9)	<0.001
	26 to 40	247 (55.0)	45 (52.9)	202 (55.5)	
	>40	143 (31.8)	17 (20)	126 (34.6)	
Child’s age (year)	<1	113 (25.2)	36 (42.3)	77 (21.2)	0.0001
	1–< 2	99 (22.0)	23 (27.1)	76 (20.9)	
	2–< 5	237 (52.8)	26 (30.6)	211 (58.0)	
Child’s birth order	First	168 (37.4)	47 (55.3)	121 (33.2)	0.0001
	Second	203 (45.2)	31 (36.5)	172 (47.3)	
	Third or higher	78 (17.4)	7 (8.2)	71 (19.5)	
Child's birth weight (gram)	<2,500	24 (5.3)	1 (1.2)	23 (6.3)	
	2,500–3,000	225 (50.1)	49 (57.6)	176 (48.4)	0.088^a^
	>3,000	200 (44.5)	35 (41.2)	165 (45.3)	
Child’s chronic comorbidity	Comorbidity	8 (1.8)	0 (0.0)	8 (2.2)	0.362^a^
	No morbidity	441 (98.2)	85 (100)	356 (97.8)	
Available vaccine	Yes	257(57.2)	46(54.1)	211(58.0)	0.518
	no	192(42.8)	39(45.9)	153(42.0)	
Experience adverse events	Yes	203(45.2)	40(47.1)	163(44.8)	0.704
	No	246(54.8)	45(52.9)	201(55.2)	
Vehicle to transport	Public transportation	12 (2.7)	1 (1.2)	11 (3.0)	0.356^a^
	Private motorbike	422 (94.0)	82 (96.5)	340 (93.4)	
	Private car	2 (0.4)	1 (1.2)	1 (0.3)	
	No vehicle	13 (2.9)	1 (1.2)	12 (3.3)	
Distance to vaccination facility	<1 km	143 (31.8)	30(35.3)	113(31.0)	0.242
	1–5 km	290 (64.6)	50(58.8)	240(65.9)	
	>5 km	16(3.6)	5(5.9)	11(3.0)	

Note:

a Fisher’s exact test.

### Bivariate analysis between parental perceptions of the EPI and vaccination adherence among minority population

Descriptive statistics of parental perceptions are provided in Table A.6/Appendix 3. Most respondents perceived susceptibility to diseases, severity of unvaccinated outcomes, and benefits of vaccination. Self-efficacy remained neutral.

All components of the HBM were included in the bivariate analysis based on the Mann-Whitney U test (Table 3). A higher mean rank in perceived barriers among non-adherent parents suggests that they experience more obstacles to vaccination. Conversely, higher mean ranks in cues to action and self-efficacy among adherent parents showed their greater motivation and confidence in vaccination adherence.

**Table 3:** Association between six components of HBM and vaccination adherence among minority population.

HBM	Mean rank		U value	*p*
	Adherence (*n* = 85)	Non-Adherence (*n* = 364)		
Perceived susceptibility	208.22	296.85	9,363.0	0.0001
Perceived severity	218.15	254.33	12,977.0	0.009
Perceived benefit	218.84	251.37	113,228.5	0.018
Perceived barriers	112.85	251.19	5,937.5	0.0001
Cues to action	258.31	217.22	12,638.5	0.007
Self-efficacy	289.19	210.01	10,013.5	0.0001

### Predictors of parental adherence to EPI for children under five years of age among minority population

The final logistic regression model included significant predictors of adherence (*p* < 0.05). Non-significant variables were excluded for interpretability.

The Log-Odds of parental vaccination adherence were calculated using the following regression equation:

Log-Odds of Adherence = 3.59 + 0.93 ∗ (Age of children under 1 years) + 1.25∗ (First birth order of children) −1.13 ∗ (Perceived barriers) + 0.61 ∗ (Self-efficacy).

Parents of children under one year were 2.54 times more likely to adhere to vaccination (aOR = 2.54, 95 % CI: 1.29–5.03) than those with children aged 24–59 months. First-born children had higher adherence (aOR = 3.48, 95 % CI: 1.36–9.92) than later-born. Greater perceived barriers were associated with lower adherence (aOR = 0.32, 95 % CI: 0.21–0.49), while higher self-efficacy increased adherence (aOR = 1.84, 95 % CI: 1.11–3.14) (Table 4).

**Table 4:** Predictors of parental adherence to EPI for children under five years of age among minority population – Binary logistic regression.

**Variables**		**B**	**aOR**^a^**(95% CI)**	***p***
Living area	Urban	0.49	1.63 (0.89–3.01)	0.11
	Rural	–	1	–
Monthly income (VND)				
	>10,000,000	0.04	1.04 (0.39–2.78)	0.93
	5000,000–10,000,000	0.19	1.21 (0.55–2.74)	0.65
	<5000,000	–	1	–
Age of participant (year)				
	≤25	0.77	2.16 (0.85–5.56)	0.11
	26 to 40	0.023	1.02 (0.48–2.22)	0.95
	>40	–	1	–
Age of children				
	<12 months	0.93	2.54 (1.29–5.03)	0.0069
	12–23 months	0.60	1.82 (0.85–3.84)	0.11
	24–59 months	–	1	–
Birth order				
	First	1.25	3.48 (1.36–9.92)	0.013
	Second	0.51	1.67 (0.66–4.64)	0.29
	Third or higher	–	1	–
Perceived susceptibility		0.61	1.83 (0.75–4.59)	0.18
Perceived severity		−0.59	0.56 (0.28–1.13)	0.097
Perceived benefit		0.14	1.15 (0.51–2.46)	0.73
Perceived barriers		−1.13	0.32 (0.21–0.49)	0.0001
Cue to action		−0.01	0.99 (0.61–1.64)	0.97
Self-efficacy		0.61	1.84 (1.11–3.11)	0.021
Constant		3.590		
AUC^b^ = 0.764				0.0001

Note:

a aOR, adjusted odds RATIO.

b AUC, area under curve.

Model fit was supported by the Hosmer-Lemeshow test (*p* = 0.094) and 82.0 % classification accuracy (Table A.3–A.4/Appendix 3). The model’s AUC was 0.86 (*p* < 0.001), indicating strong discrimination. See Table A.5 and Figure A.2 (Appendices 3–4) for full classification performance.

## Discussion

A key component of public health initiatives to stop vaccine-preventable diseases is making sure that everyone is vaccinated on time and completely. However, achieving high vaccination compliance rates remains a challenge in underprivileged communities. This study underscored the importance of assessing not just vaccination coverage but also adherence to schedules to achieve comprehensive population immunity. Despite Vietnam's high vaccination coverage rates, adherence among ethnic minority communities remained low, with only 18.9 % of parents adhering to recommended schedules. This discrepancy exposed a gap in the effectiveness of the EPI in addressing the needs of underserved populations.

The existence of global and regional disparities in adherence might reveal as follows: the adherence rates among ethnic minorities in Vietnam were lower compared to similar studies conducted among Arab communities in Israel, where DPT/Hib and measles adherence rates were 92 % and 82 %, respectively.^22^ Higher adherence rates in Israel have been linked to supportive societal norms, universal health coverage, and robust follow-up and pediatric services. In comparison, the 56.7 % adherence rate among migrant children on the Thailand–Myanmar border was notably higher than the 18.9 % among ethnic minority children in Vietnam. This may reflect Thailand’s more proactive policies and mobile immunization programs targeting hard-to-reach and undocumented populations.^6^ In contrast, Vietnam’s EPI in border areas still relies heavily on fixed-site delivery, with limited outreach strategies.

In addition to parental decisions, healthcare infrastructure plays a critical role in vaccination adherence. Children in areas with better access to hospital deliveries and community prenatal care had significantly higher rates of timely immunization.^5^ Seasonal agricultural work may also cause delays, as parents deprioritize vaccination visits. In rural minority areas, children often receive delayed rather than refused immunizations—most are eventually vaccinated, but adherence to the schedule remains challenging.^5^

Adherence further declines as children age, consistent with findings from the Korean CDC.^23^ Parents may initially follow schedules closely but become less vigilant as children grow, especially if they appear healthy or have previously fallen ill despite vaccination.^23^ Older children are also more likely to have comorbidities, which can complicate vaccination.^24^ Other factors—such as fear of needles.^25^ past side effects.^26^ and competing demands—can also reduce adherence.

Age-related declines may reflect gaps in health system outreach. While newborns benefit from routine postpartum contacts, booster doses (e.g., JE2, JE3) often rely on parental initiative. This reduced engagement by the health system over time may lead to missed or delayed vaccinations. Given Vietnam’s cumulative EPI schedule, older children face more chances of falling behind. Our strict adherence definition—timely for all vaccines—may lower adherence rates in older age groups, not due to reduced parental effort but due to longer exposure to potential delays.

Firstborn children showed higher adherence rates than later-born siblings, consistent with findings from multiple countries.^27^ Parents often prioritize the health needs of their first child and follow medical advice more strictly, while in larger families, divided attention and increased childcare experience may reduce the urgency for subsequent children.^28^ Health centers should implement targeted reminder systems for these higher-risk families to support timely vaccination.

HBM analysis identified perceived barriers—such as time constraints, vaccine unavailability, and limited information—as key obstacles to adherence, echoing studies from South Korea and Saudi Arabia.^29^,^30^ In Saudi Arabia, high self-efficacy was sometimes linked to lower adherence due to exposure to anti-vaccine narratives and distrust in vaccine safety.^30^ In contrast, our study found that higher self-efficacy was associated with better adherence, as confident parents trusted local health workers and the EPI system.

This study's predictive model (binary logistic regression) reliably identified families at risk of non-adherence to the EPI using demographic (child’s age, birth order) and psychosocial factors (perceived barriers, self-efficacy). Health workers can apply priority screening to guide follow-up visits, targeted counseling, reminder systems, or educational outreach. This approach allows for efficient resource allocation by focusing on families most likely to miss or delay vaccinations.

Several limitations should be noted. First, without survey weighting, adherence estimates may not represent all communes equally, affecting generalizability. Second, while vaccination cards were verified, self-reported data on socio-demographics, parental perceptions, vaccine provision, service access, and child characteristics may introduce recall bias. Third, clustering effects were not adjusted for; though households were randomly selected, unmeasured commune-level factors (e.g., infrastructure, outreach programs) may have influenced outcomes. Future studies should consider cluster-adjusted analyses to account for these contextual variables. Lastly, future models may be improved by including factors such as parental trust in healthcare, knowledge of the immunization schedule, and exposure to vaccine misinformation.

## Conclusion

Parental adherence to the EPI for children under five in Dong Thap’s minority population was low (18.9 %). Key predictors included perceived barriers, self-efficacy, child age, and birth order. Interventions should prioritize older and higher birth order children and address logistical and psychological barriers such as access, time constraints, and concerns about adverse events.

Strengthening collaboration with local leaders and health volunteers and using multilingual education materials can improve outreach and confidence. Integrating vaccination with maternal-child health visits, along with SMS reminders and home visits, may support timely immunization in mobile populations. In cross-border areas, robust tracking systems are vital due to migration challenges. Training healthcare workers in culturally sensitive communication and involving experienced parents in peer-support programs can further strengthen adherence efforts.

## Authors contributions

An Dai Tran (30%) led the conceptualization, data collection, and drafting of the manuscript. Cua Ngoc Le (30%) supervised the study, performed data analysis, and provided critical revisions.

Charuai Suwanbamrung (5%) and Muhammad Haroon Stanikzai (3%) assisted with methodology development and literature review.

Nirachon Chutipattana (5%) and Shamarina Shohaimin (5%) supported data validation and formatting.

Patthanasak Khammaneechan (5%) and Le Minh Luan (5%) contributed to data interpretation and manuscript editing.

Tran Phu Dien (5%), Truong Thanh Nam (5%), and Phan Thanh Tung (2%) participated in field coordination and data acquisition.

## Conflicts of interest

The authors declare no conflicts of interest.

## Appendix Supplementary materials

Image, application 1
