# Supplementary material for: Parental predictors of childhood vaccination adherence in border areas of Southern Vietnam: a first look at minority communities
Source: J Pediatr (Rio J). 2025 May 21;101(4):642–50. doi: 10.1016/j.jped.2025.04.005 (PMC12276618; doi:10.1016/j.jped.2025.04.005)
Supplement: Supplementary file 1 [file mmc1.docx]

**JPED-D-24-00618_Supplementary Materials**

**Appendix 1**

**Questionnaire**

This questionnaire aims to study the level of adherence to the expanded vaccination program of ethnic minority parents to under-five-years children in Dong Thap province. Please answer the following questions by filling out the blanks below. Your answer will bring great benefits to understanding the context of adherence to the expanded program on immunization among ethnic minority parents in Dong Thap province. The information you provide in this questionnaire will be kept confidential. Your information will not be disclosed. The information will be used for research purposes only. In case you have any questions, please contact:

Name: An Dai Tran

Address 1: School of Public Health, Walailak University, 222 Thaiburi, Tha Sala District, Nakhon Si Thamarat, Thailand.

Address 2: Dong Thap Provincial Center for Disease Control, 394 Le Dai Hanh, My Phu Ward, City. Cao Lanh, Dong Thap province

Phone number: 0824143768 Email: [daiandt2016@gmail.com](mailto:daiandt2016@gmail.com)

| Time | Day …… Month ..…. Year 2024  Collector’s name :…………….. | Participant ID:  …………………………………… |
| --- | --- | --- |
| Location | City/District…………………………….………………………………..  Commune………………………………………………………………..  Hamlet…………………………………………………………………… | |

This questionnaire includes questions divided into three parts as follows:

Part 1: Socio-demographic characteristics of parents (interviewees)

Part 2: Parental perception of vaccination

Part 3: Vaccine provision

Part 4: Accessibility of health service

Part 5: Demographic characteristics of children

Part 6: Parental Adherence to Expanded Program on Immunization

|  | | |
| --- | --- | --- |
| PART 1: Socio-demographic characteristics of parental (Please check (✓) the box) | | |
| A1 | What is kinship between you and children in your family?  ☐Father ☐ Grandparents  ☐ Mother ☐ Sibling  ☐ Relative | |
| A2 | Gender  ☐ 1. Male ☐ 2. Female | |
| A3 | Which area do you currently live in?  ☐ 1. Urban ☐ 2. Rural | |
| A4 | Number of family members: ………………………………………………………... | |
| A5 | Your current marital status is:  ☐ 1. Have a family.  ☐ 2. Married but currently in a state of separation/divorced.  ☐ 3. Single  *Note: If you choose Option 1, the interviewer will be gathering information about both the husband and wife, from question A6 to question A9.* | |
| A6 | Your year of birth:  …………………… | Year of birth of your husband/wife: ……………………………….. |
| A7 | What is your highest level of education? | What is your husband/wife highest level of education? |
|  | ☐ 1. Not going to school  ☐ 2. Primary (grades 1-5)  ☐ 3. Junior high school (grades 6-9)  ☐ 4. Senior high school (grades 10-12)  ☐ 5. Associate degree  ☐ 6. Bachelor’s degree or upper | ☐ 1. Not going to school  ☐ 2. Primary (grades 1-5)  ☐ 3. Junior high school (grades 6-9)  ☐ 4. Senior high school (grades 10-12)  ☐ 5. Associate degree  ☐ 6. Bachelor’s degree or upper |
| A8 | What is your average monthly income?  ………………………(VND) | What is your husband/wife average monthly income?  …………………………………(VND) |
| A9 | What is your current career?  ☐ 1. Already have a stable job  (Specify: ……………………….)  ☐ 2. Freelance, seasonal work  ☐ 3. Unemployed  ☐ 4. Housewife  ☐ 5. Students  ☐ 6. Retirement  ☐ 6. Farmer  ☐ 8. Other  (Specify……………………………….) | What is your husband/wife current career?  ☐ 1. Already have a stable job  (Specify: …………………………....)  ☐ 2. Freelance, seasonal work  ☐ 3. Unemployed  ☐ 4. Housewife  ☐ 5. Students  ☐ 6. Retirement  ☐ 6. Farmer  ☐ 8. Other  (Specify………………………………..) |

| PART 2 | Parental perception of vaccination. | | | | | |
| --- | --- | --- | --- | --- | --- | --- |
|  | Read each sentence and tick (✓) in the appropriate box. There is no right or wrong answer. And don’t dwell too long on any one sentence.  *Degree evaluation:*  1. “strongly disagree”  2. “disagree”  3. “not sure”  4. “agree”  5. “strongly agree” | | | | | |
|  |  | (1) | (2) | (3) | (4) | (5) |
|  | Perceived susceptibility |  |  |  |  |  |
| B1 | You mean your children will be susceptible to diseases if they do not get their vaccinations on schedule | ☐ | ☐ | ☐ | ☐ | ☐ |
| B2 | Vaccinating children is very important | ☐ | ☐ | ☐ | ☐ | ☐ |
| B3 | If other children in your household or surrounding area have a disease (measles, pertussis, diphtheria, tetanus, polio, tuberculosis, or Japanese encephalitis B), your child is at risk of contracting the disease. | ☐ | ☐ | ☐ | ☐ | ☐ |
|  | Perceived severity |  |  |  |  |  |
| B4 | It will be dangerous if a child gets sick (such as measles, pertussis, diphtheria, tetanus, polio, tuberculosis, Japanese encephalitis B) | ☐ | ☐ | ☐ | ☐ | ☐ |
| B5 | Suppose a child suffers from diseases (such as measles, pertussis, diphtheria, tetanus, polio, tuberculosis, and Japanese encephalitis B). He/she will suffer from the sequelae of the disease | ☐ | ☐ | ☐ | ☐ | ☐ |
|  | Perceived benefits |  |  |  |  |  |
| B6 | The expanded program on immunization protects your children from diseases (like measles, pertussis, diphtheria, tetanus, polio, tuberculosis, and Japanese B encephalitis) | ☐ | ☐ | ☐ | ☐ | ☐ |
| B7 | The Expanded Program on Immunization helps parents reduce the time spent caring for their children when they fall ill. | ☐ | ☐ | ☐ | ☐ | ☐ |
| B8 | Vaccinating your child will also help prevent other children from getting sick. | ☐ | ☐ | ☐ | ☐ | ☐ |
|  | Perceived barriers |  |  |  |  |  |
| B9 | Adverse event might happen after children received vaccine | ☐ | ☐ | ☐ | ☐ | ☐ |
| B10 | I do not have enough time to take my child for vaccination | ☐ | ☐ | ☐ | ☐ | ☐ |
| B11 | The vaccination schedule has too many vaccines, which led to you missing out on some vaccines. | ☐ | ☐ | ☐ | ☐ | ☐ |
| B12 | The Medical staff are not friendly. Health officials do not provide enough information on the efficacy and side effects of each vaccine. | ☐ | ☐ | ☐ | ☐ | ☐ |
| B13 | My house is far from the medical facilities. | ☐ | ☐ | ☐ | ☐ | ☐ |
| B14 | Medical facilities do not have vaccines yet. | ☐ | ☐ | ☐ | ☐ | ☐ |
| B15 | Comorbidities of children at the time point at vaccination that will delay vaccination | ☐ | ☐ | ☐ | ☐ | ☐ |
|  | Cues to action |  |  |  |  |  |
| B16 | The medical staff told me that vaccination is good for my child and that I should get vaccinated. | ☐ | ☐ | ☐ | ☐ | ☐ |
| B17 | The medical center informed me that vaccination is good for my child, I should get vaccinated | ☐ | ☐ | ☐ | ☐ | ☐ |
| B18 | Information on TV, social networks like Facebook, TikTok, YouTube... say that vaccination is good for my child, I should get vaccinated. | ☐ | ☐ | ☐ | ☐ | ☐ |
|  | Self-efficacy |  |  |  |  |  |
| B19 | You won’t be influenced by others’ negative attitude about vaccination | ☐ | ☐ | ☐ | ☐ | ☐ |
| B20 | You can effectively influence others’ trust on vaccination. | ☐ | ☐ | ☐ | ☐ | ☐ |
| B21 | You often actively seek about vaccination information by yourself | ☐ | ☐ | ☐ | ☐ | ☐ |

| PART 3 | Vaccine provision |
| --- | --- |
| C1 | When you take your child to a vaccination facility, are the required vaccines available for administration?  ☐Yes  ☐ No |
| C2 | Do your children have experienced adverse events or serious adverse events?  ☐Yes  ☐ No |
| PART 4: | Accessibility of health service |
| D1 | What is the vehicle of transport that you use to take your child to the vaccination facility?  ☐public transport (bus, taxi)  ☐ Private motorbike  ☐ Private car  ☐ Motorbike taxi |
| D2 | How many kilometers is the distance from your house to the facility?  ………………………………………………………………………………………………….. |
| PART 5 | Demographic characteristics of children |
| E1 | Date of birth ………………………………………. |
| E2 | Who takes care of the child most of the time?  ☐Father ☐ Grandparents  ☐ Mother ☐ Sibling  ☐ Relative |
| E3 | Birth order  ☐ First ☐ Second ☐ Third ☐ Fourth ☐ Fifth |
| E4 | Where is the child's birth place?  ☐ At home ☐ Hospital/health center☐ Village health station  ☐ Other (specific……………………………………………………………..) |
| E5 | Birth weight of the child:………………………………………………....) |
| E6 | Chronic comorbidity  ☐ Congenital heart  ☐ Asthma  ☐ Autoimmune diseases  ☐ Strong reaction to vaccines  ☐ Other (Specific………………………………………………………………………) |

| Part 6: Parental adherence to Expanded Program on Immunization | | | | | | | | | | | | | | |
| --- | --- | --- | --- | --- | --- | --- | --- | --- | --- | --- | --- | --- | --- | --- |
|  | Age of children | Vaccines | | | | | | | | | Check Received Date | | | |
| N^o^ |  | BCG | HBV | DPT-Hep B-Hib | OPV | IPV | Measles | MR | DPT | Japanese encephalitis B | Date | In time | Delay | No vaccination |
| F1 | At birth | Dose 1 |  |  |  |  |  |  |  |  | _ _ _ _ _ | ☐ | ☐ | ☐ |
| F2 |  |  | Birth dose |  |  |  |  |  |  |  | _ _ _ _ _ | ☐ | ☐ | ☐ |
| F3 | 2 months |  |  | Dose 1 |  |  |  |  |  |  | _ _ _ _ _ | ☐ | ☐ | ☐ |
| F4 |  |  |  |  | Dose 1 |  |  |  |  |  | _ _ _ _ _ | ☐ | ☐ | ☐ |
| F5 | 3 months |  |  | Dose 2 |  |  |  |  |  |  | _ _ _ _ _ | ☐ | ☐ | ☐ |
| F6 |  |  |  |  | Dose 2 |  |  |  |  |  | _ _ _ _ _ | ☐ | ☐ | ☐ |
| F7 | 4 months |  |  | Dose 3 |  |  |  |  |  |  | _ _ _ _ _ | ☐ | ☐ | ☐ |
| F8 |  |  |  |  | Dose 3 |  |  |  |  |  | _ _ _ _ _ | ☐ | ☐ | ☐ |
| F9 | 5 months |  |  |  |  | Dose 1 |  |  |  |  | _ _ _ _ _ | ☐ | ☐ | ☐ |
| F10 | 9 months |  |  |  |  |  | Dose 1 |  |  |  | _ _ _ _ _ | ☐ | ☐ | ☐ |
| F11 | 12 months |  |  |  |  |  |  |  |  | Dose 1 | _ _ _ _ _ | ☐ | ☐ | ☐ |
| F12 | 12.5 months |  |  |  |  |  |  |  |  | Dose 2 | _ _ _ _ _ | ☐ | ☐ | ☐ |
| F13 | 18 months |  |  |  |  |  |  | Dose 1 |  |  | _ _ _ _ _ | ☐ | ☐ | ☐ |
| F14 | 18 months |  |  |  |  |  |  |  | Dose1 |  | _ _ _ _ _ | ☐ | ☐ | ☐ |
| F15 | 2 to 5 years |  |  |  |  |  |  |  |  | Dose3 | _ _ _ _ _ | ☐ | ☐ | ☐ |

**Appendix 2**

List of variables and their categorization

| \| **Variable Name** \| \| --- \| | \| **Questionnaire Item** \| \| --- \| | \| **Variable Type** \| \| --- \| | **Categorization** |
| --- | --- | --- | --- | --- | --- | --- |
| **PART 1: Socio-demographic characteristics ofparental** | | | |
| Kinship to Child | What is your kinship with the child? | **Categorical** | Father, Mother, Grandparent, Sibling, Relative |
| Gender | Gender | **Categorical** | Male, Female |
| Residence area | Which area do you currently live in? | **Categorical** | Urban, Rural |
| Household size | Number of family members | **Continuous** | Numerical value (open-ended) |
| Marital Status | Current marital status | \| **Categorical** \| \| --- \| | \| 1. Married,  2. Separated/Divorced,  3. Single \| \| --- \| |
| Year of Birth | Your year of birth | \| **Continuous** \| \| --- \| | \| Numerical (Year) \| \| --- \| |
| Spouse’s Year of Birth | Year of birth of your husband/wife | \| **Continuous** \| \| --- \| | Numerical (Year) |
| Education Level | What is your highest level of education? | **Ordinal** | 1. No School, 2. Primary, 3. Junior High, 4. Senior High, 5. Associate, 6. Bachelor’s or Higher |
| Spouse’s Education Level | What is your husband/wife ‘s highest level of education? | **Ordinal** | 1. No School, 2. Primary, 3. Junior High, 4. Senior High, 5. Associate, 6. Bachelor’s or Higher |
| Monthly Income | What is your average monthly income? | \| **Continuous** \| \| --- \| | Numerical (VND) |
| Spouse’s Monthly Income | What is your husband/wife ‘s average monthly income? | \| **Continuous** \| \| --- \| | Numerical (VND) |
| Occupation | What is your current career? | \| **Categorical** \| \| --- \| | \| 1. Stable Job, 2. Freelance/Seasonal, 3. Unemployed,  4. Housewife,  5. Student, 6. Retired,  7. Farmer, 8. Other \| \| --- \| |
| Spouse’s Occupation | What is your husband/wife’s current career? | \| **Categorical** \| \| --- \| | \| 1. Stable Job, 2. Freelance/Seasonal, 3. Unemployed,  4. Housewife,  5. Student, 6. Retired,  7. Farmer, 8. Other \| \| --- \| |
| **PART 2:** Parental perception of vaccination | | | |
| Perceived Susceptibility | You believe your children will be susceptible to diseases if they do not get vaccinated on schedule. | Ordinal | 5-point Likert scale (1 = Strongly Disagree, 5 = Strongly Agree). |
|  | Vaccinating children is very important. | Ordinal | 5-point Likert scale (1 = Strongly Disagree, 5 = Strongly Agree). |
|  | If other children in the household or nearby have a disease, your child is at risk of contracting it. | Ordinal | 5-point Likert scale (1 = Strongly Disagree, 5 = Strongly Agree). |
| Perceived Severity | It will be dangerous if a child contracts a vaccine-preventable disease. | Ordinal | 5-point Likert scale (1 = Strongly Disagree, 5 = Strongly Agree). |
|  | A child who suffers from vaccine-preventable diseases will experience long-term health consequences. | Ordinal | 5-point Likert scale (1 = Strongly Disagree, 5 = Strongly Agree). |
| Perceived Benefits | The Expanded Program on Immunization (EPI) protects your children from vaccine-preventable diseases. | Ordinal | 5-point Likert scale (1 = Strongly Disagree, 5 = Strongly Agree). |
|  | The EPI helps parents reduce the time spent caring for sick children. | Ordinal | 5-point Likert scale (1 = Strongly Disagree, 5 = Strongly Agree). |
|  | Vaccinating your child will also help prevent other children from getting sick. | Ordinal | 5-point Likert scale (1 = Strongly Disagree, 5 = Strongly Agree). |
| Perceived Barriers | Adverse events might occur after vaccination. | Ordinal | 5-point Likert scale (1 = Strongly Disagree, 5 = Strongly Agree). |
|  | I do not have enough time to take my child for vaccination. | Ordinal | 5-point Likert scale (1 = Strongly Disagree, 5 = Strongly Agree). |
|  | The vaccination schedule has too many vaccines, leading to missed doses. | Ordinal | 5-point Likert scale (1 = Strongly Disagree, 5 = Strongly Agree). |
|  | Medical staff are not friendly or do not provide enough information about vaccines. | Ordinal | 5-point Likert scale (1 = Strongly Disagree, 5 = Strongly Agree). |
|  | My house is far from the medical facilities. | Ordinal | 5-point Likert scale (1 = Strongly Disagree, 5 = Strongly Agree). |
|  | Medical facilities do not always have vaccines available. | Ordinal | 5-point Likert scale (1 = Strongly Disagree, 5 = Strongly Agree). |
|  | My child’s comorbidities at the time of vaccination may delay their immunization. | Ordinal | 5-point Likert scale (1 = Strongly Disagree, 5 = Strongly Agree). |
| Cues to Action | A medical staff member told me that vaccination is good for my child and that I should vaccinate them. | Ordinal | 5-point Likert scale (1 = Strongly Disagree, 5 = Strongly Agree). |
|  | The medical center informed me that vaccination is good for my child, and I should vaccinate them. | Ordinal | 5-point Likert scale (1 = Strongly Disagree, 5 = Strongly Agree). |
|  | Information from TV, social media (Facebook, TikTok, YouTube) supports vaccination. | Ordinal | 5-point Likert scale (1 = Strongly Disagree, 5 = Strongly Agree). |
| Self-Efficacy | I am not influenced by others' negative attitudes toward vaccination. | Ordinal | 5-point Likert scale (1 = Strongly Disagree, 5 = Strongly Agree). |
|  | I can effectively influence others' trust in vaccination. | Ordinal | 5-point Likert scale (1 = Strongly Disagree, 5 = Strongly Agree). |
|  | I actively seek vaccination information on my own. | Ordinal | 5-point Likert scale (1 = Strongly Disagree, 5 = Strongly Agree). |
| **PART 3:** Vaccine Provision | | | |
| \| **Vaccine**  **Availability** \| \| --- \| | \| When you take your child to a vaccination  facility, are the required vaccines  available? \| \| --- \| | \| **Categorical** \| \| --- \| | \| Yes, No \| \| --- \| |
| \| **Adverse**  **Events** \| \| --- \| | \| Has your child experienced any adverse  or serious adverse events after vaccination? \| \| --- \| | **Categorical** | \| Yes, No \| \| --- \| |
| **PART 4:** Accessibility of Health Service | | | |
| Mode of Transportation | What type of transport do you use to take your child to the vaccination facility? | Categorical | Public transport, Private motorbike, Private car, Motorbike taxi |
| \| **Distance to**  **Facility** \| \| --- \| | \| How many kilometers is the distance  from your house to the vaccination facility? \| \| --- \| | \| **Continuous** \| \| --- \| | \| Numerical value  (open-ended) \| \| --- \| |
| **PART 5 Demographic characteristics of children** | | | |
| \| **Child’s Date of**  **Birth** \| \| --- \| | \| Date of birth \| \| --- \| | \| **Continuous** \| \| --- \| | \| Date (DD/MM/YYYY) \| \| --- \| |
| \| **Primary**  **Caregiver** \| \| --- \| | \| Who takes care of the child most  of the time? \| \| --- \| | \| **Categorical** \| \| --- \| | \| Father, Mother,  Grandparents, Sibling,  Relative \| \| --- \| |
| \| **Birth Order** \| \| --- \| | \| Birth order of the child \| \| --- \| | \| **Categorical** \| \| --- \| | \| First, Second, Third,  Fourth, Fifth \| \| --- \| |
| \| **Place of Birth** \| \| --- \| | \| Where was the child born? \| \| --- \| | \| **Categorical** \| \| --- \| | \| Home, Hospital/Health  center, Village health  station, Other (Specify) \| \| --- \| |
| \| **Birth Weight** \| \| --- \| | \| What was the child's birth weight? \| \| --- \| | \| **Continuous** \| \| --- \| | \| Weight in grams/kilograms \| \| --- \| |
| \| **Chronic**  **Comorbidities** \| \| --- \| | \| Does the child have any chronic  comorbidities? \| \| --- \| | \| **Categorical** \| \| --- \| | \| Congenital heart, Asthma,  Autoimmune diseases,  Strong reaction to vaccines,  Other (Specify) \| \| --- \| |

**Appendix 3**

**Table A.1** Economic-demographic characteristics of study participants.

| Economic-demographic characteristics | | Frequency (n)  (n = 449) | Percentage  (%) |
| --- | --- | --- | --- |
| Kinship between participant with children | Father | 41 | 9.1 |
|  | Mother | 228 | 64.1 |
|  | Relative | 7 | 1.6 |
|  | Grandparents | 112 | 24.9 |
|  | Sibling | 1 | 0.2 |
| Gender | Male | 62 | 13.8 |
|  | Female | 387 | 86.2 |
| Living area | Urban | 143 | 31.8 |
|  | Rural | 306 | 68.2 |
| Family member count | ≤ 4 | 182 | 40.5 |
|  | > 4 | 267 | 59.5 |
| Marital status | Married | 425 | 94.7 |
|  | Separated/divorced | 20 | 4.5 |
|  | Single | 4 | 0.9 |
| Education level | Below senior high school | 300 | 66.8 |
|  | Senior high school and above | 149 | 33.2 |
| Income | < 5,000,000 VND | 123 | 27.4 |
|  | 5,000,000 to 10,000,000 VND | 238 | 53.0 |
|  | > 10,000,000 VND | 88 | 19.6 |
| Career | Month salary job | 54 | 12.0 |
|  | Freelance, seasonal work | 88 | 19.6 |
|  | Unemployed | 1 | 0.2 |
|  | Home maker | 199 | 44.3 |
|  | Student | 69 | 15.4 |
|  | Retired | 21 | 4.7 |
|  | Farmer | 17 | 3.8 |
| Age | ≤ 25 | 59 | 13.1 |
|  | 26 to 40 | 248 | 55.2 |
|  | > 40 | 142 | 31.6 |
| Age | Mean ± SD  (Median) | 37.86  (34.00) | |

**Table A.2** Demographic characteristics of children in the study population.

| Demographic characteristics of children | | Frequency (n)  n = 449 | Percentage (%) |
| --- | --- | --- | --- |
| Main caregiver | Father | 20 | 4.5 |
|  | Mother | 309 | 68.8 |
|  | Relative | 8 | 1.8 |
|  | Grandparents | 111 | 24.7 |
|  | Sibling | 1 | 0.2 |
| Age | < 1 age | 113 | 25.2 |
|  | 1 to less than 2 ages | 99 | 22.0 |
|  | 2-5 ages | 237 | 52.8 |
| Birth order | First | 168 | 37.4 |
|  | Second | 203 | 45.2 |
|  | Third or higher | 78 | 17.4 |
| Birthplace | At home | 2 | 0.4 |
|  | Hospital/health center | 442 | 98.4 |
|  | Village health station | 5 | 1.1 |
| Weight at the birth | < 2500 gram | 24 | 5.3 |
|  | 2500-3000 gram | 225 | 50.1 |
|  | >3000 gram | 200 | 44.5 |
| Chronic comorbidity | Comorbidity | 8 | 1.8 |
|  | Non-morbidity | 441 | 98.2 |

**Table A.3** Hosmer and Lemeshow Goodness-of-Fit Test Results.

| **Model** | **Chi Square** | **df** | **p-value** |
| --- | --- | --- | --- |
| Binary logistic regression | 13.772 | 8 | 0.094 |

**Table A.4** Overall classification accuracy of the predicting binary logistic regression model.

| **Observed** | **Predicted** | | |
| --- | --- | --- | --- |
|  | **Non-adherence** | **Adherence** | **Percentage Correct** |
| Non-adherence | 340 | 24 | 93.4 |
| Adherence | 57 | 28 | 32.9 |
| Overall percentage |  |  | 82.0 |

**Table A.5** Threshold Analysis for Sensitivity and Specificity based on ROC Curve.

| **Threshold Probability** | **Sensitivity** | **1 - Specificity** | **Distance =** $\sqrt{\left( \left( \boldsymbol{1-sensitivity} \right)^{\boldsymbol{2}}\boldsymbol{+}\left( \boldsymbol{1-specificity} \right)^{\boldsymbol{2}} \right)}$ |
| --- | --- | --- | --- |
| 0.19566 | 0.788 | 0.223 | 0.30769 |
| 0.19516 | 0.788 | 0.225 | 0.309142 |
| 0.19395 | 0.788 | 0.228 | 0.311333 |
| 0.19237 | 0.788 | 0.231 | 0.313536 |
| 0.1906 | 0.788 | 0.234 | 0.315753 |
| 0.19586 | 0.776 | 0.223 | 0.316078 |
| 0.20223 | 0.765 | 0.212 | 0.316495 |
| 0.18949 | 0.788 | 0.236 | 0.317238 |
| 0.20019 | 0.765 | 0.214 | 0.317838 |
| 0.21688 | 0.753 | 0.201 | 0.318449 |
| 0.18921 | 0.788 | 0.239 | 0.319476 |
| 0.21453 | 0.753 | 0.203 | 0.319715 |
| 0.19799 | 0.765 | 0.217 | 0.319866 |
| 0.23177 | 0.741 | 0.19 | 0.321218 |
| 0.21184 | 0.753 | 0.206 | 0.321629 |
| 0.18792 | 0.788 | 0.242 | 0.321727 |
| 0.19685 | 0.765 | 0.22 | 0.321908 |
| 0.23067 | 0.741 | 0.192 | 0.322405 |
| 0.20871 | 0.753 | 0.209 | 0.323558 |
| 0.19609 | 0.765 | 0.223 | 0.323966 |
| 0.18537 | 0.788 | 0.245 | 0.323989 |
| 0.2285 | 0.741 | 0.195 | 0.324201 |
| 0.18159 | 0.788 | 0.247 | 0.325504 |
| 0.20483 | 0.753 | 0.212 | 0.325504 |
| 0.22666 | 0.741 | 0.198 | 0.326014 |
| 0.17848 | 0.788 | 0.25 | 0.327787 |
| 0.22235 | 0.741 | 0.201 | 0.327844 |
| 0.23296 | 0.729 | 0.187 | 0.329257 |
| 0.1761 | 0.788 | 0.253 | 0.33008 |
| 0.23248 | 0.729 | 0.19 | 0.33097 |
| 0.17385 | 0.788 | 0.255 | 0.331616 |
| 0.17305 | 0.788 | 0.258 | 0.333928 |

**Table A.6** Descriptive Statistics of Parental Perceptions of the Expanded Program on Immunization (EPI).

| \| **HBM Component** \| \| --- \| | \| **Mean** \| \| --- \| | \| **Standard Deviation (SD)** \| \| --- \| |
| --- | --- | --- | --- | --- | --- |
| Perceived Susceptibility | 4.12 | 0.48 |
| Perceived Severity | 4.01 | 0.52 |
| Perceived Benefits | 4.26 | 0.41 |
| Perceived Barriers | 3.77 | 0.83 |
| Cues to Action | 3.68 | 0.66 |
| Self-Efficacy | 3.45 | 0.70 |

**Table A.7** Vaccination type and doses recommended by Vietnam’s EPI.[18]

| **Age of Children** | **Vaccine Type** | **Cumulative Doses Expected** |
| --- | --- | --- |
| At birth | HBV, BCG _(a)_ | HBV: 1 dose, BCG: 1 dose |
| 2 months | HBV, BCG, DPT-Hep B-Hib_(b)_, OPV_(c)_ | HBV: 1 dose, BCG: 1 dose, DPT-Hep B-Hib: 1 dose, OPV: 1 dose |
| 3 months | HBV, BCG, DPT-Hep B-Hib, OPV | HBV: 1 dose, BCG: 1 dose, DPT-Hep B-Hib: 2 doses, OPV: 2 doses |
| 4 months | HBV, BCG, DPT-Hep B-Hib, OPV | HBV: 1 dose, BCG: 1 dose, DPT-Hep B-Hib: 3 doses, OPV: 3 doses |
| 5 months | HBV, BCG, DPT-Hep B-Hib, OPV, IPV _(d)_ | HBV: 1 dose, BCG: 1 dose, DPT-Hep B-Hib: 3 doses, OPV: 3 doses, IPV: 1 dose |
| 9 months | HBV, BCG, DPT-Hep B-Hib, OPV, IPV, MCV _(e)_ | HBV: 1 dose, BCG: 1 dose, DPT-Hep B-Hib: 3 doses, OPV: 3 doses, IPV: 1 dose, MCV: 1 dose |
| 12 months | HBV, BCG, DPT-Hep B-Hib, OPV, IPV, MCV, JE _(f)_ | HBV: 1 dose, BCG: 1 dose, DPT-Hep B-Hib: 3 doses, OPV: 3 doses, IPV: 1 dose, MCV: 1 dose, JE: 1 dose |
| 12 months & 2 weeks | HBV, BCG, DPT-Hep B-Hib, OPV, IPV, MCV, JE | HBV: 1 dose, BCG: 1 dose, DPT-Hep B-Hib: 3 doses, OPV: 3 doses, IPV: 1 dose, MCV: 1 dose, JE: 2 doses |
| 18 months | HBV, BCG, DPT-Hep B-Hib, OPV, IPV, MCV, JE,  MR _(g)_ , DPT_(h)_ | HBV: 1 dose, BCG: 1 dose, DPT-Hep B-Hib: 3 doses, OPV: 3 doses, IPV: 1 dose, MCV: 2 doses, JE: 2 doses, MR: 1 dose, DPT: 1 booster dose |
| 2 to 5 years | HBV, BCG, DPT-Hep B-Hib, OPV, IPV, MCV, JE, MR, DPT _(h_ | HBV: 1 dose, BCG: 1 dose, DPT-Hep B-Hib: 3 doses, OPV: 3 doses, IPV: 1 dose, MCV: 3 doses, JE: 3 doses, MR: 1 dose, DPT: 1 booster dose |

Note: (a) HBV, Hepatitis-B vaccine; BCG, Bacillus Calmette-Guérin; (b) DPT-Hep B-Hib, Diphtheria, Pertussis, Tetanus, Hepatitis B, Haemophilus influenzae type b; (c) OPV, Oral Poliomyelitis vaccine; (d) IPV, Inactivated polio vaccine; (e) MCV, measles-containing vaccine; (f) JE, Japanese encephalitis; (g) MR, Measles, rubella; (h) DPT, Diphtheria, Pertussis, Tetanus.

**Table A.8** Content validity of items in the questionnaire from part 1 to 5.

| **Items/ questionnaire** | **Expert 1** | **Expert 2** | **Expert 3** | **I-CVI** |
| --- | --- | --- | --- | --- |
| Part 1: Socio-demographic characteristics ofparental |  |  |  |  |
| A1 | 4 | 4 | 4 | 1 |
| A2 | 4 | 4 | 4 | 1 |
| A3 | 4 | 4 | 4 | 1 |
| A4 | 4 | 4 | 4 | 1 |
| A5 | 4 | 4 | 4 | 1 |
| A6 | 4 | 4 | 4 | 1 |
| A7 | 4 | 4 | 4 | 1 |
| A8 | 4 | 4 | 4 | 1 |
| A9 | 4 | 4 | 4 | 1 |
| Part 2: Parental perception of the EPI |  |  |  |  |
| B1 | 4 | 4 | 4 | 1 |
| B2 | 4 | 4 | 4 | 1 |
| B3 | 4 | 4 | 4 | 1 |
| B4 | 4 | 4 | 4 | 1 |
| B5 | 4 | 4 | 4 | 1 |
| B6 | 4 | 4 | 4 | 1 |
| B7 | 4 | 4 | 4 | 1 |
| B8 | 4 | 4 | 4 | 1 |
| B9 | 4 | 4 | 4 | 1 |
| B10 | 4 | 4 | 4 | 1 |
| B11 | 4 | 4 | 4 | 1 |
| B12 | 4 | 4 | 4 | 1 |
| B13 | 4 | 4 | 4 | 1 |
| B14 | 4 | 4 | 4 | 1 |
| B15 | 4 | 4 | 4 | 1 |
| B16 | 4 | 4 | 4 | 1 |
| B17 | 4 | 4 | 4 | 1 |
| B18 | 4 | 4 | 4 | 1 |
| B19 | 4 | 4 | 4 | 1 |
| B20 | 4 | 4 | 4 | 1 |
| B21 | 4 | 4 | 4 | 1 |
| Part 3. Vaccine provision |  |  |  |  |
| C1 | 3 | 4 | 4 | 1 |
| C2 | 3 | 4 | 4 | 1 |
| Part 4. Accessibility of health service |  |  |  |  |
| D1 | 2 | 4 | 4 | 0.67 |
| D2 | 2 | 4 | 4 | 0.67 |
| Part 5. Demographic characteristics of children |  |  |  |  |
| E1 | 4 | 4 | 4 | 1 |
| E2 | 3 | 4 | 4 | 1 |
| E3 | 2 | 4 | 4 | 0.67 |
| E4 | 2 | 4 | 4 | 0.67 |
| E5 | 3 | 4 | 4 | 1 |
| E6 | 3 | 4 | 4 | 1 |
| S-CVI/Ave = 0.96  S-CVI/UA = 0.9 | | | | |

To ensure the measurement quality, validated content and reliable scales were adopted. Three experts assessed the questionnaire’s content validity. The item-level Content Validity Index (I-CVI) was calculated by dividing the number of experts who rated an item as 3 or 4 by the total number of experts.[20] The scale-level CVI average (S-CVI/Ave) was determined by averaging the I-CVI scores across items, while the universal agreement scale-level CVI (S-CVI/UA) reflected the percentage of items rated 3 or 4 by all experts. For the research questionnaire, S-CVI/Ave and S-CVI/UA of at least 0.80 were considered acceptable.[21]

**Appendix 4**

**Figure A.1** Parental adherence to EPI by age of children.


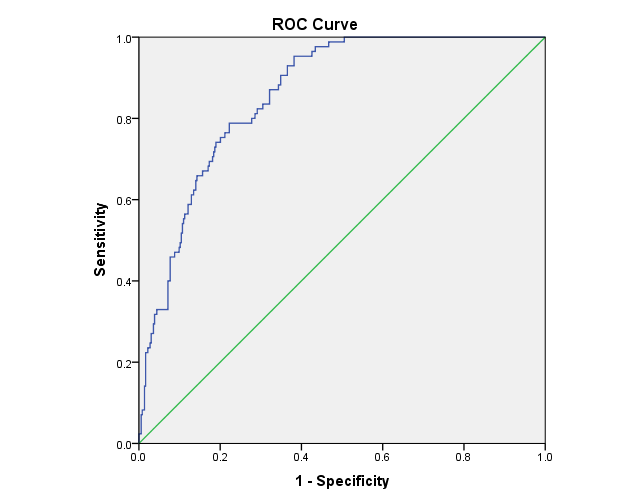


**Figure A.2** Receiver Operating Characteristic (ROC) curve for the predicted probability of the binary logistic regression model in predicting parental vaccination adherence. The ROC curve is positioned close to the top-left corner, significantly distant from the 45-degree diagonal, further demonstrating model accuracy. These findings provide a quantifiable method for distinguishing between adherent and non-adherent parents based on the survey.
